# Incomplete and delayed Sox2 deletion defines residual ear neurosensory development and maintenance

**DOI:** 10.1038/srep38253

**Published:** 2016-12-05

**Authors:** Martina Dvorakova, Israt Jahan, Iva Macova, Tetyana Chumak, Romana Bohuslavova, Josef Syka, Bernd Fritzsch, Gabriela Pavlinkova

**Affiliations:** 1 Institute of Biotechnology CAS, Prague, Czechia; 2 Faculty of Science, Charles University, Prague, Czechia; 3 Department of Biology, University of Iowa, Iowa City, IA, USA; 4 Institute of Experimental Medicine CAS, Prague, Czechia

## Abstract

The role of Sox2 in neurosensory development is not yet fully understood. Using mice with conditional *Islet1-cre* mediated deletion of *Sox2*, we explored the function of Sox2 in neurosensory development in a model with limited cell type diversification, the inner ear. In *Sox2* conditional mutants, neurons initially appear to form normally, whereas late- differentiating neurons of the cochlear apex never form. Variable numbers of hair cells differentiate in the utricle, saccule, and cochlear base but sensory epithelium formation is completely absent in the apex and all three cristae of the semicircular canal ampullae. Hair cells differentiate only in sensory epithelia known or proposed to have a lineage relationship of neurons and hair cells. All initially formed neurons lacking hair cell targets die by apoptosis days after they project toward non-existing epithelia. Therefore, late neuronal development depends directly on Sox2 for differentiation and on the survival of hair cells, possibly derived from common neurosensory precursors.

SRY-box (Sox) transcription factors are essential for embryonic and adult neuronal stem cell development^1^. Sox2 and other Sox genes dynamically specify the proliferating neuronal lineage, upregulate the differentiating transcription of basic helix-loop-helix (bHLH) factors and are ultimately downregulated by bHLH transcription factors to allow the differentiation of neurons and glia cells^2^ in sequential transcriptional waves^3^. This process is conserved across neurosensory evolution^4^ but is difficult to assess in the brain due to the complexity of a multitude of cell fate decisions. The developing inner ear has just two neurosensory cell types: hair cells (HCs) for mechanotransduction and sensory neurons to conduct information to the brain^5^,^6^. The ear provides a simpler model to study Sox2 involvement in specification, proliferation, and differentiation compared to the brain.

The mammalian inner ear has six sensory epithelia: Five vestibular (the maculae of utricle and saccule, and the cristae of the three semicircular canal ampullae) and the organ of Corti, the auditory sensory organ of the cochlea. Inner ear neuronal development primarily depends on *Neurogenin1 (Neurog1*) and *Neurod1* for neuronal specification and differentiation. Sensory fate specification occurs after neurogenesis and requires the expression of *Atoh1*^7^,^8^,^9^. The expression of these bHLH transcription factors requires additional signaling molecules to regulate the transition from active proliferation to a progenitor cell state primed for commitment and differentiation. In particular, Sox2 may play an important role in this transition during inner ear development. The very early expression of Sox2 through the otic placode overlaps with the earliest forming neurons^7^,^10^ and it is expressed in both neurogenic and sensory progenitors^11^. Sox2 may also be involved in non-sensory development^12^. Experimental data indicate that there are complex antagonistic and cooperative interactions between Sox2 and bHLH transcription factors during the development of the inner ear. Sox2 activates *Atoh1* and *Neurog1,* but also promotes negative regulators of *Atoh1* and *Neurog1* like *Neurod1*, thereby suppressing premature HC and neuronal differentiation in mice and chickens^13^,^14^. The results in chickens^15^ suggest that Sox2 is part of a regulatory network responsible for the specification of neuronal *versus* prosensory cell fates in inner ear development. However, the role of Sox2 in the transcriptional network that controls neurosensory development in mice, beyond promoting it, remains unclear.

To analyze the molecular effects of *Sox2* deletion on the neurosensory development of the ear, we generated a new mouse model of *Sox2* conditional deletion, *Islet1-cre;Sox2*^*f/f*^ (*Isl1-cre;Sox2*^*f/f*^). We chose *Isl1-cre* to achieve a partial overlap of *cre* and *Sox2* expression in the sensory epithelium and delaminating neurons in the inner ear^11^,^12^,^16^,^17^. The delayed elimination of Sox2 by Isl1-cre does not affect the earliest specification events in the developing ear, as these *Isl1-cre;Sox2*^*f/f*^ mutants show an initial formation of vestibular neurons and spiral ganglion (SG) neurons in the cochlear base. In contrast, SG neurons, which differentiate last, never form in the apex. Additionally, conditional mutant mice do not develop cristae of the semicircular canal ampullae and show correlated semicircular canal defects. In contrast, the utricle, saccule and base of the cochlea develop a variable number of HCs receiving an aberrant innervation from the few surviving neurons. All initially formed neurons lacking hair cell targets die by apoptosis days after they project toward non-existing epithelia. In summary, our data indicate that some HCs are refractory to the delayed loss of Sox2 and neuronal formation and viability depends on Sox2 both directly and indirectly.

## Results

### *Sox2, Isl1* and *Isl1-cre* are partially co-expressed in the otocyst

To analyze the molecular effects of *Sox2* deletion on the neurosensory development of the inner ear, we generated a new mouse model of *Sox2* conditional deletion using *Isl1-cre/*+ with a direct *cre* knockin into the endogenous *Isl1* locus^18^. First, we characterized Isl1 and Isl1-cre protein early expression in the otocyst at E9.5 and found that their expression patterns overlap (Supplementary Fig. 1a). Isl1 and Isl1-cre were expressed in the delaminated vestibular neurons and also within the otic neurosensory epithelium at E9.5 (Supplementary Fig. 1a’,a”). Similarly, the cells co-expressing Isl1 and Sox2 were detected in the otic epithelium as early as E9.5 (Supplementary Fig. 1a”, arrows). However, Isl1 and Isl1-cre are not as widely expressed as Sox2 in the early sensory epithelium of the otic vesicle. Next, we analyzed the cre-mediated excision within the developing inner ear using a R26R-*lacZ* reporter line and *in situ* hybridization for *Isl1* mRNA. At E10.5, cre-mediated β-gal reporter expression showed cre recombination in the tissues corresponding to the expression of *Isl1* mRNA and protein (Supplementary Fig. 1b–c’). *Isl1* mRNA, cre, and protein are expressed in the progenitor cells of the sensory epithelium and in delaminating sensory neurons, consistent with previously reported data^16^,^17^.

### Conditional deletion of Sox2 causes inner ear dysmorphology

Heterozygous *Isl1-cre/*+*;Sox2*^*f/*+^ mice are viable and can be used as breeding stock. The morphology of the inner ear and hearing functions were comparable to wild type mice (Supplementary Fig. 2). The decreased gene dose of *Sox2* in heterozygous *Isl1-cre/*+*;Sox2*^*f/*+^ mice did not affect the normal development and maintenance of the sensory organs of the inner ear in any detectable way. In contrast, *Isl1-cre/*+*;Sox2*^*f/f*^mutants (Sox2 CKO) died immediately after birth, although they did not exhibit any external morphological defects beyond a reduced and misshapen eye. These data demonstrate that *Isl1-cre* can be used for the recombination of *Sox2*^*f/f*^ in common precursors for sensory epithelia and sensory neurons and that homozygous mice develop a severe enough phenotype that they die at birth.

**Figure 1:**
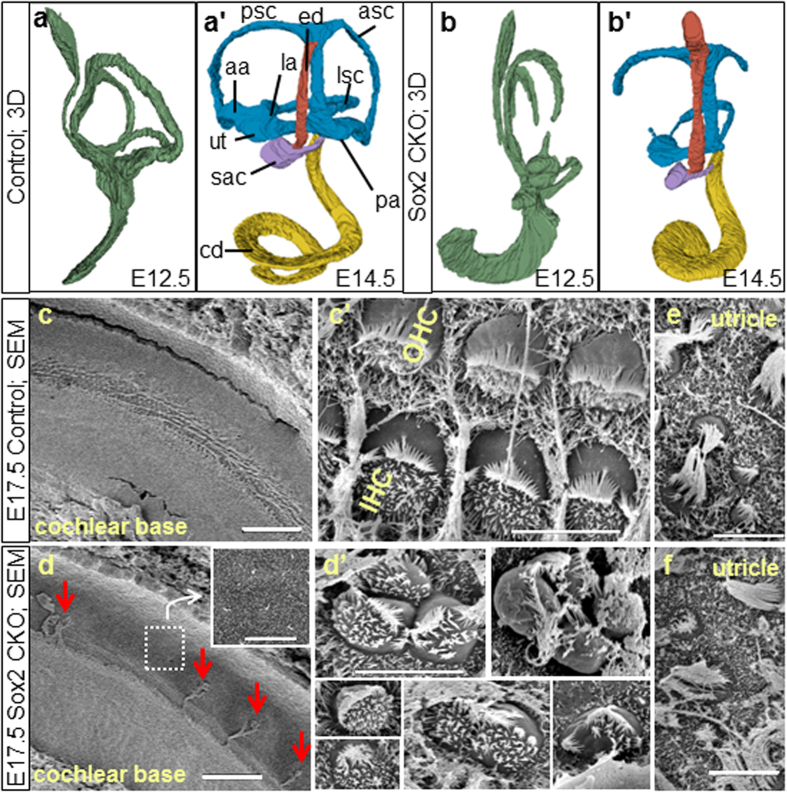
Altered morphology of the Sox2 CKO inner ear. (**a-b’**) 3D-reconstruction reveals severe changes in the developing inner ear at E12.5 and E14.5. (**b,b’**) No ampullae of semicircular canals and only rudiments of the posterior and anterior semicircular canals are present in Sox2 CKO. The utricle and saccule are smaller. (**a’,b’**) The cochlear duct (cd) has decreased coiling and is shorter compared to controls. (**c**–**f**) Scanning electron microscopy shows a few individual cells and small clumps of cells with a hair cell-like phenotype in the base of the Sox2 CKO cochlea (arrows). (**d**) The rest of the organ of Corti is missing as shown by the overview of the whole cd width and by magnification of the sensory epithelium area. (**d’**) HCs vary in size, orientation and bundle organization. (**e**,**f**) The cellular phenotype of differentiated HCs in the Sox2 CKO utricle is comparable to controls. aa, anterior ampulla; asc, anterior semicircular canal; cd, cochlear duct; ed, endolymphatic duct; la, lateral ampulla; lsc, lateral semicircular canal; pa, posterior ampulla; psc, posterior semicircular canal; sac, saccule; ut, utricle; OHC, outer hair cells; IHC, inner hair cells. Scale bars: 50 μm (c,d), 5 μm (c’,d’,e,f).

A computer assisted 3D-reconstruction revealed profound differences in the ear at E12.5 and E14.5 (Fig. 1a-b’). The *Sox2* CKO inner ears had no semicircular canal ampullae or cristae of the ampullae, and only rudiments of the posterior and anterior semicircular canals with the horizontal canal being the only canal ever forming. The formation of a horizontal canal is consistent with previous reports that this canal can form in the absence of a crista^19^, whereas the formation of the vertical canals depends on Fgf10/BMP4 interactions expressed in the cristae of the anterior and posterior vertical canal ampullae^20^. The utricle and saccule were smaller, and the cochlear duct was 20% shorter compared to controls at E14.5. This phenotype was comparable to previous reports on the *Lcc* (light coat and circling; a mutant generated by a X-ray-irradiation-induced mutation with a severe inner ear malformation due to the absence of Sox2 in the developing inner ear)^21^. Scanning electron microscopy showed a profound reduction of sensory epithelia in Sox2 CKO. Only individual HCs or clusters of HCs formed in the basal half of the cochlea with undifferentiated epithelial cells between them (Fig. 1d), while the spiral sulcus and tectorial membrane formed normally. HCs in the Sox2 CKO cochlea differed in their size, orientation, and bundle organization (Fig. 1d’). There was no sign of HC differentiation in the entire apex, instead a flat epithelium was observed, as previously described for the *Lcc* mutant^21^. Differentiated HCs in the utricle in terms of cell size and stereocilia formation were comparable between Sox2 CKO and controls (Fig. 1e,f).

### Incomplete and delayed Isl1-cre recombination of floxed *Sox2* alters inner ear gene expression

We next investigated how rapidly Sox2 protein disappears after *Isl1-cre* expression. E10.5 ears already showed a reduced overlap of Isl1 and Sox2 immunostaining in the Sox2 CKO, indicating a loss of Sox2^+^ cells in the neurosensory epithelium (Supplementary Fig. 3a,b). Some detectable Sox2 protein may be found in the Sox2 CKO ear at E11.5 (Supplementary Fig. 3b’). At E13.5, Sox2 was expressed throughout the entire length of the control cochlea, whereas, in the Sox2 CKO cochlea, Sox2 was detected only in the base (Supplementary Fig. 3c,d). Therefore, we next investigated how rapidly *Sox2* mRNA disappears after Isl1-cre mediated recombination (Supplementary Fig. 3e–h). These data show a surprisingly uneven loss of *Sox2* expression in the Sox2 CKO: whereas no *Sox2* signal was detectable in all three cristae of the semicircular canal ampullae, the utricle showed a minor, and the saccule and cochlea a more obvious, reduction at E11.5 (Supplementary Fig. 3f,f’). At E13.5, there was no expression of *Sox2* mRNA in the cristae of the semicircular canal ampullae and the apex of the cochlea, limited expression in the base of the cochlea, and weak expression in the utricle and saccule (Supplementary Fig. 3h) compared to controls (Supplementary Fig. 3g). Notably, neither at E11.5 nor at E13.5 was there any expression of *Sox2* in all three cristae of the semicircular canal ampullae or apical half of the growing cochlear duct. These data suggest that Isl1-cre rapidly and effectively recombines floxed *Sox2* in certain areas of Isl1 expression but not so in others. As Sox2 expression precedes Isl1-expression, this suggests delayed Isl1-cre recombination. Using Isl1-cre to delete *Atoh1*^*f/f*^ (Atoh1 CKO), we showed that Isl1-cre has a uniform effect in all sensory epithelia of the inner ear with a complete loss of HCs (Supplementary Fig. 4). As opposed to Pax2-cre deletion of *Atoh1*^22^, Isl1-cre deletion of *Atoh1* shows no residual Sox2 expression in sensory epithelia and concomitant innervation. Atoh1 CKO data combined with Pax2-cre *Atoh1* data suggest a simple delayed recombination of *Sox2* by Isl1-cre in the epithelium.

Since *Sox2* is completely lost in all three cristae of the semicircular canal ampullae (Supplementary Fig. 3f) and cristae formation critically depends on Fgf10 for normal development^23^, we next tested the expression of *Fgf10* mRNA (Supplementary Fig. 3i,j). No canal crista labeling for *Fgf10* could be detected and late developmental stages showed limited *Fgf10* labeling in some saccular neurons of Sox2 CKO. These data imply an unexpected level of dependency of Fgf10 on Sox2. How direct or indirect this effect is, is yet to be determined.

### Biphasic loss of neurons indicates direct and indirect effects of Sox2 CKO

**Figure 2:**
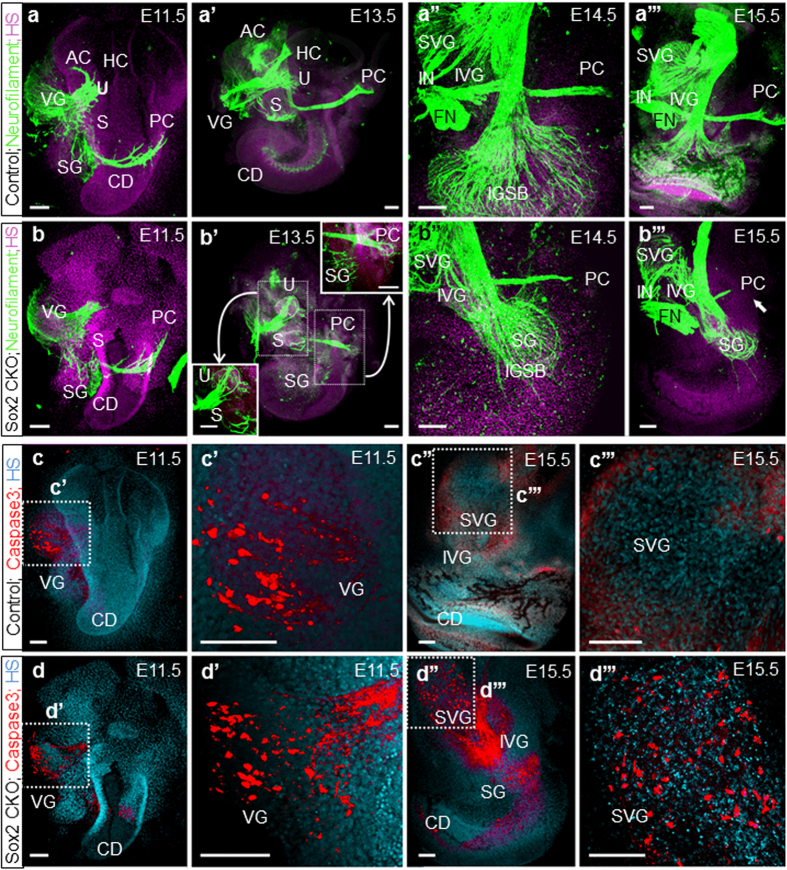
Isl1-cre mediated Sox2 loss disrupts neuron formation and results in massive neuronal degeneration by activation of Caspase3. (**a,b**) Immunofluorescence staining of neurofilament in the Sox2 CKO shows similar formation of vestibular neurons at E11.5 compared to controls. (**a’,b’**) At E13.5, fibers are aberrantly projecting toward the utricle (or combined utricle and anterior and horizontal canal cristae) and posterior canal cristae of the Sox2 CKO. (**a”,b”,a”’,b”’**) Fibers to the posterior canal crista start to retract in the absence of target HCs starting at E14.5 in the mutant. (**b”’**) Only a few radial fibers are formed near the base of the E15.5 Sox2 CKO cochlea. (**c, c’,d,d’**) Immunofluorescence of activated Caspase3 reveals positive staining restricted mainly in the VG in the E11.5 Sox2 CKO comparable to the control littermates. (**c”,c”’,d”,d”’**) However, Caspase3 mediated cell death is massively progressed to IVG, SVG, and SG at E15.5 compared to no caspase positive cells in the control littermates. Scale bars: 100 μm. AC, anterior canal crista; CD, cochlear duct; FN, facial nerve; HS, Hoechst nuclear stain; IGSB, intraganglionic spiral bundle; IVG, inferior vestibular ganglion; IN, intermediate nerve; HC, horizontal canal crista; PC, posterior canal crista; S, saccule; SG, spiral ganglion; SVG, superior vestibular ganglion; U, utricle; VG, vestibular ganglia.

The expression of Neurog1 and neuronal delamination is among the earliest specification events in the developing ear^7^,^24^. The initial onset of Sox2 expression is nearly simultaneous with Neurog1 specifying neurogenic progenitors in the otic placode as early as E8.5^25^. One day later at E9.5, Isl1 is expressed in the delaminated vestibular neurons and in the neuroblast precursor cells within the otic epithelium^17^. Consistent with expression patterns, the early neurogenic specification events were unaffected by Isl1-cre mediated *Sox2* recombination, as demonstrated by a comparable formation of the delaminated vestibular neurons between Sox2 CKO and controls at E10.5 (Supplementary Fig. 3a,b). Furthermore, the expression of early neuronal differentiation marker, Neurod1, was comparable between controls and Sox2 CKO at E11.5, which is consistent with normal neuron differentiation (Supplementary Fig. 3k,l). Similarly, in the E11.5 inner ear, there was no obvious change in the expression of Pax2 (Supplementary Fig. 3m,n), one of the earliest genes expressed during inner ear development^26^. Neurofilament immunocytochemistry showed a limited growth of fibers toward the region of the anterior and horizontal semicircular canal crista in the Sox2 CKO inner ear, whereas growth toward the utricle, saccule, and posterior canal crista was similar between Sox2 CKO and controls (Fig. 2a,b). Later stages had fibers that did not innervate the ‘crista organ region’ but formed loops in the area where these organs should have been (Fig. 2a’’), which is similar to previous records on Fgf10 mutants^23^. These fibers of cristae of the semicircular canal ampullae remained until E14.5 (Fig. 2a””), when they regressed and disappeared nearly completely by E18.5, leaving only a variable innervation to the utricle and saccule (Supplementary Fig. 6c–f). Our data suggest that many vestibular neurons form, develop, and project but are only later eliminated. Consistent with the progressive loss of vestibular fibers, we found increased Caspase 3 immunolabeling in the vestibular ganglion neurons in older stages (Fig. 2c-d”’), indicating rapid apoptosis of most vestibular neurons, likely due to the lack of neurotrophins^27^ and/or HCs^22^. Quantification of Caspase 3 positive cells (E11.5–15.5) revealed a significant increase of Caspase 3 in the Sox2 CKO mutant (Supplementary Fig. 5). SG neurons form only near the base (Fig. 2b,b”’, Supplementary Fig. 6b,b’) but never in the apex of the mutant. Fewer basal SG neurons are present after E15.5, and by E18.5 only a few neurons are left projecting toward the base and occasionally sending fibers toward the apex (Supplementary Fig. 6e). Since only basal turn SG neurons ever form, we also found Caspase 3 immunocytochemical signals only in the base at E15.5 (Fig. 2d”), indicating a loss of initially formed SG neurons due to the absence of sensory epithelium.

### Delayed deletion of *Sox2* allows limited HC differentiation in some epithelia

**Figure 3:**
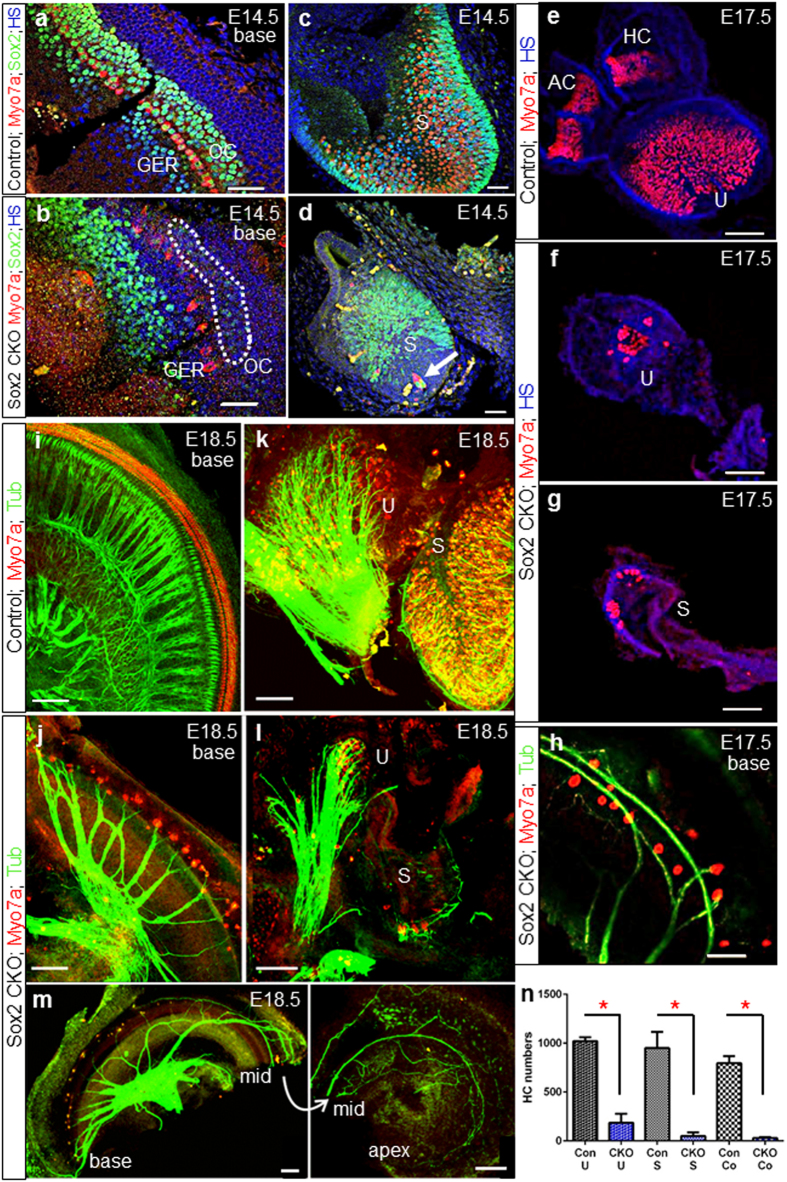
Delayed deletion of Sox2 results in the differentiation of some neurosensory cells in the basal cochlear turn and in the vestibular organs. (**a,b**) Sox2^+^ cells in the Sox2 CKO cochlea are detected only in the base at the age of E14.5 and disappear later in development. (**b**) The strong Sox2 expression domain is shifted toward the GER. Similarly, Myo7a^+^ cells do not differentiate in the proper area of OC. Some weak Sox2 expression remains in the OC area of Sox2 CKO (dotted area). (**c,d**) Variable numbers of HCs (Myo7a^+^) and supporting cells (Sox2^+^) develop in the Sox2 CKO vestibular system. (**d**) HCs in the saccule also develop in the area that lacks supporting cells (arrow). (**e–h**) Some poorly differentiated Myo7a^+^ HCs are present in the utricle, saccule and basal turn of the cochlea of the Sox2 CKO at E17.5. (**i–m**) At E18.5, the innervation of mutant cochlea, saccule and utricle is severely reduced and shows an unusual pattern compared to controls. Fibers show mostly directional growth toward remaining HCs but also transient expansion into HC-free regions. (**n**) The quantification of Myo7a positive HCs after whole mount immunostaining shows a striking reduction of HCs in the Sox2 CKO inner ear compared to littermate controls for the utricle (U), saccule (S) and cochlea (Co). Myo7a^+^ HCs were counted after whole mount immunostaining using LAS AF Lite draw counter to avoid counting error. The total number of HCs was determined in the entire utricle and saccule, and in the entire Sox2 CKO cochlea. The number of HCs in the control cochlea represents the total number of HCs in 1.5 mm of the base. The values represent means ± SD (N = 4–7 individuals/group). *P < 0.0001, *t*-test. Scale bars: 50 μm (a–h), 100 μm (i–m). AC, anterior crista; GER, greater epithelial ridge; HC, horizontal crista; OC, organ of Corti; S, saccule; U, utricle.

To define the extent of cellular changes in the inner ear of Sox2 CKO, the expression of specific markers for neuronal innervation (tubulin) and HCs (Myo7a) was analyzed. The Sox2 CKO sensory epithelium of the utricle, saccule, and cochlear base was smaller with a reduced domain of Sox2^+^ at E14.5 (Fig. 3a–d, Supplementary Fig. 7). No Sox2 protein was ever detected in the apex despite transient labeling of the base (Fig. 3b, Supplementary Fig. 3d). In the cochlear base, the strong Sox2 expression domain was shifted toward the greater epithelial ridge (GER), whereas scattered cells with a very limited Sox2 expression were detected in the organ of Corti (Fig. 3b, dotted area). Consistent with the residual expression of Sox2 in the CKO utricle, saccule, and basal turn of the cochlea, we found Myo7a positive HCs only in these epithelia (Fig. 3e–l). We never found any Myo7a positive cells in the apex of the cochlea or cristae of the semicircular canal ampullae. This suggests that a limited amount of Sox2 expression for a yet to be determined time is needed to maintain and differentiate HC precursors into HCs. A complete deletion of Sox2 in the cristae of the semicircular canal ampullae and the apex leads to a complete loss of all HC differentiation. We next quantified the number of Myo7a positive cells and found that the utricle and saccule showed a profound variability (Fig. 3n, Supplementary Fig. 8, Table S1). Detailed quantification using Myo7a as a HC marker revealed a reduction of HCs even in the best cases and in most cases down to around 10% of the control littermates.

### The remaining differentiated HCs dictate the residual pattern of innervation

The patterning of the inner ear, and in particular the innervation of the cochlea, requires a multitude of known and unknown molecules to guide the nerve fibers to the sensory epithelia and to sort the pattern of innervation to the distinct cell types within the sensory epithelia^6^,^28^. The absence of Sox2 seems to have no effect on the initial growth of fibers that is apparently primarily directed along Schwann cells^29^. Since only scattered HCs remain, an unusual pattern of innervation emerges in all sensory epithelia with fibers showing directional growth toward remaining HCs but also transient expansion into HC-free territories like the apex (Fig. 3m). In the basal turn of the organ of Corti, spiraling fibers may be found that are directed toward the apex (Fig. 3j,m, Supplementary Fig. 6) and not the base, as is typically the case for type II SGN fibers^6^,^30^. In general, the remaining HCs were the target of residual innervation, but fibers could overshoot and extend into HC-free territory, suggesting guiding issues possibly related to the Sox2 deletion in the neurons and/or supporting cells. A similar phenotype of fibers extending into HC-free territory was reported in mice with a loss of Schwann cells^29^. The variation in the number and distribution of the remaining HCs, combined with the transient viability of neurons outside HC areas, apparently determines the variation in the pattern of residual innervation.

### Differentiated HCs in the Sox2 CKO inner ear have unusual features

**Figure 4:**
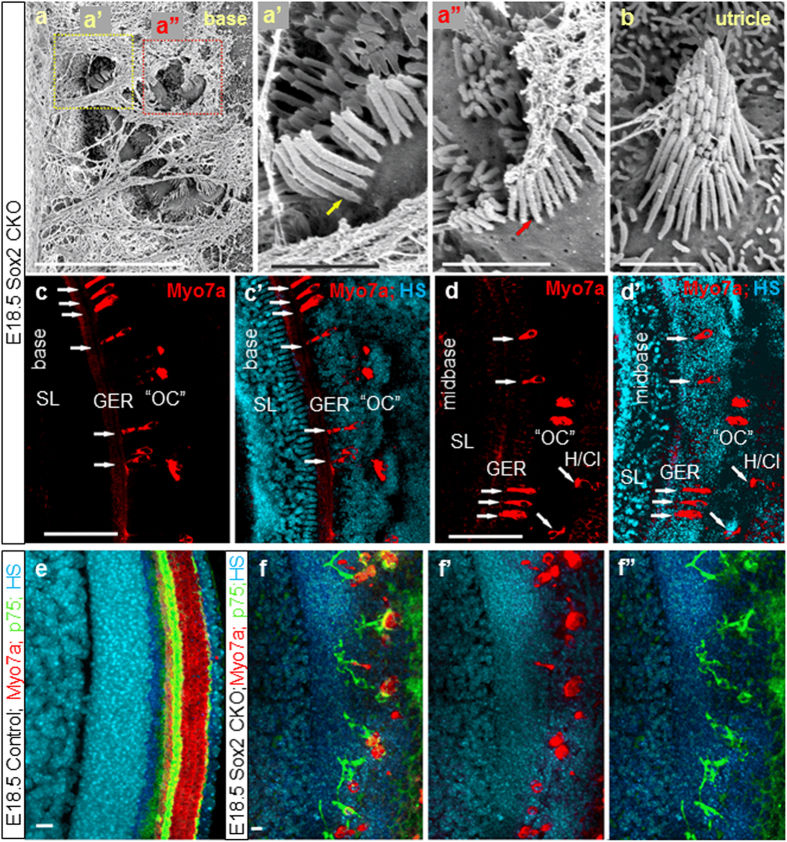
Aberrant HCs in ectopic topology and abnormal pillar cells are formed in the Sox2 CKO. Patches of HCs in the base of the E18.5 Sox2 CKO cochlea are covered by a tectorial membrane with HCs in the topology of inner HCs displaying both large diameter (**a,a’**) and small diameter (**a,a”**) stereocilia reminiscent of inner and outer HCs, respectively. (**b**) Vestibular HCs show normal organization of stereocilia but many display variability in stereocilia diameter in a single HC, normally associated with either type I or type II vestibular HCs. (**c-d’**) Scattered Myo7a positive HCs are detected in the area corresponding topologically to the organ of Corti (OC); however, forming atypical organ of Corti (“OC”) in the mutant. Immunostaining of Myo7a reveals formation of HCs in the ectopic topologies, medial to OC, in the GER, as well as lateral to OC (in the area of Hensen/Claudius cells) (white arrows) in addition to the area of “OC” in the E18.5 Sox2 CKO. (**e-f”**) The combination of p75 and Myo7a immunolabeling shows an unusual configuration and distribution of p75 positive cells near the remaining Myo7a positive HCs in E18.5 Sox2 CKO compared to the single row of p75^+^ inner pillar cells in control littermates (**e**). Scale bars: 10 μm (a,e-f”), 1 μm (a’,a”,b), 100 μm (c-d’). GER, greater epithelial ridge; H/Cl, Hensen/Claudius cells; OC, organ of Corti; “OC”, atypical organ of Corti in the mutant; SL, spiral limbus.

Closer inspection of epithelia with a higher number of Myo7a positive cells revealed a variable degree of differentiation of HCs in terms of stereocilia development, cell size, distribution, and viability (Fig. 1, Fig. 4a-a”). HCs differentiate as distinct types recognizable by their stereocilia with a complete absence of innervation^7^,^31^. Four distinct types of HCs are found in the mammalian inner ear with respect to stereocilia arrangement and stereocilia diameters (type I + II vestibular HCs, inner and outer HCs of the cochlea). In fact, inner and outer HCs are identified (as the name implies) based on their position relative to pillar cells. In the vestibular organs, type I and type II hair cells are known to be distinct based on the stereocilia bundle^32^. Furthermore, cochlea stereocilia bundles are very different from those of vestibular organs. While the molecular components determining the polarity within a given HC and across sensory epithelia are emerging^33^,^34^, there is limited evidence on the molecular basis of vestibular versus cochlear HC bundle organization or the distinct differences in stereocilia diameters of inner and outer HCs^30^. Sox2 seems to play a minor role in this process, as many differentiating HCs in the Sox2 CKO have the sensory-epithelium-specific polarity and bundle organization. While stereocilia specific to all four types of HCs were found in the Sox2 CKO inner ear, some HCs showed a mixed diameter of stereocilia, indicating an incomplete segregation of the two vestibular and cochlear HC types after transient and limited expression of Sox2 (Fig. 4a-a”). Whether this is a direct effect of Sox2 or is related to the previously described effect of the level of Atoh1 in cochlear HC differentiation^35^ remains to be seen.

**Figure 5:**
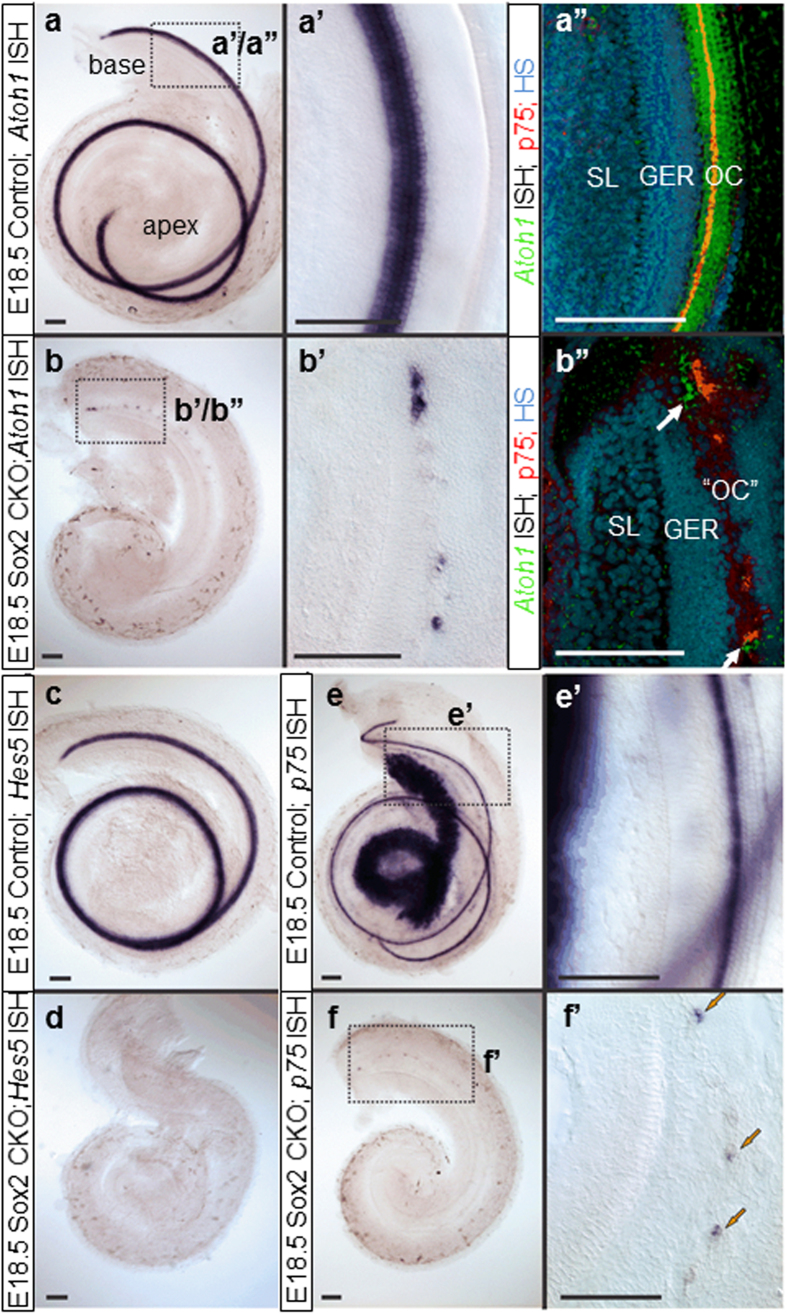
Loss of Sox2 affects downstream gene expression in the organ of Corti. (**a,a’,b,b’**) *Atoh1* expression is dramatically reduced in Sox2 CKO mice at E18.5. (**a”,b”**) *Atoh1* ISH signal converting into a fluorescent signal shows the relative topology to the inner pillar cell marker p75. Note that the scattered HCs are found medial and lateral to p75 positive cells (white arrows; b”). However, p75 expression (arrows) is discontinuous in the base (**b”,f,f’**) and absent in the apex compared to prominent labeling in the inner pillar cells and the spiral ganglion neurons in control animals (**e,e’**). (**c**,**d**) Another supporting cell marker, *Hes5*, shows no expression at all in the Sox2 CKO mice at E18.5. Scale bars: 100 μm. GER, greater epithelial ridge; OC, organ of Corti; “OC”, atypical organ of Corti in the mutant; SL, spiral limbus.

We also found many ectopic Myo7a positive HCs in GER, as well as in the area of Hensen/Claudius cells (lateral to the organ of Corti) in the Sox2 CKO cochlear base (Fig. 4c-d’). The combination of p75 and Myo7a immunolabeling shows an unusual configuration and distribution of p75 positive cells near the remaining Myo7a positive HCs in E18.5 Sox2 CKO compared to the single row of p75^+^ inner pillar cells in control littermates (Fig. 4e-f”). Nuclear staining revealed apoptosis in many HCs, suggesting that the variability in numbers is driven by two processes: the reduced formation of viable HCs and the loss of some unviable HCs. Longitudinal studies of Sox2 protein levels in viable and non-viable HCs are needed to clarify how early Sox2 protein levels define HC viability. Since both the level^34^ and duration of Atoh1 expression^36^ determine the normal differentiation of HCs, we investigated the expression of *Atoh1* using *in situ* hybridization^33^. Compared to the profound labeling of control littermates, we only found a limited, patchy expression of *Atoh1* in the basal turn of the cochlea (Fig. 5a-b”), corresponding to the expression of Sox2 in the base of the Sox2 CKO. These results confirm that *Atoh1* expression and the possible level of expression critically depend on Sox2. The dose effect of Sox2 in activating the transcription of *Atoh1* has previously been shown in cochlear explant cultures^37^. Correspondingly, as shown by the HC marker (Myo7a), the HCs formed small clusters only in the basal turn of the cochlea (Fig. 3). The Sox2 CKO phenotype confirms that *Atoh1* expression and subsequent HC differentiation depends on Sox2, because *Atoh1* expression and differentiated HCs were detected only in the cochlear base of the Sox2 CKO, where Sox2 is initially expressed. However, the unusual cluster pattern and features of these differentiated HCs suggest that the induction of *Atoh1* expression and subsequent HC differentiation require a specific level and duration of Sox2 expression.

**Figure 6:**
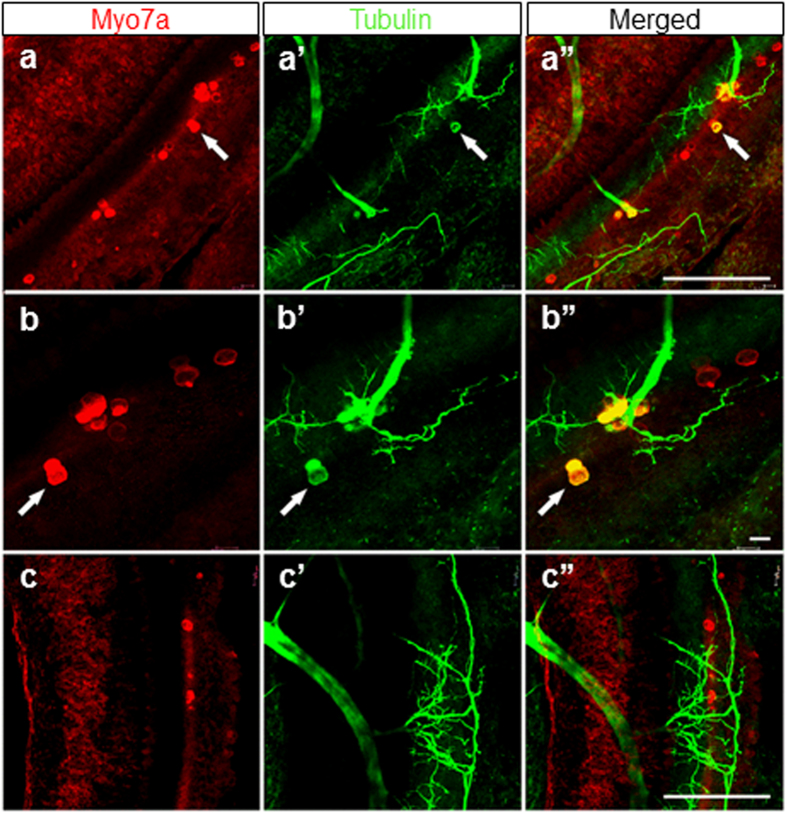
Some HCs are positive for both Myo7a and tubulin. These single or groups of Myo7a positive HCs of E18.5 Sox2 CKO mice show a patchy distribution (**a**,**b**,**c**) and an unusual pattern of innervation (**a’,b’,c’**). Note that most fibers are targeted toward Myo7a positive HCs, others are sometimes widely distributed in the topological equivalent of the organ of Corti. (**a”,b”,c”**) Some Myo7a positive cells are also positive for antibody directed against tubulin, normally a reliable neuronal marker in the ear. Scale bars: 100 μm, except b,b” that indicates 10 μm.

In the ear, HCs are normally negative for tubulin, which serves as a neuronal marker, even when neurons are experimentally converted to HCs^14^. In the Sox2 CKO, however, many remaining HCs showed positive staining for tubulin in addition to the HC marker, Myo7a (Fig. 6). Sox2 levels and timing of expression may therefore also play a role in the segregation of neuronal and HC phenotype that is incomplete for some HCs in our conditional mutant.

## Discussion

Our conditional deletion of *Sox2* using Isl1-cre provides the first mouse model that tests *in vivo* the function of Sox2 in inner ear sensory neuron formation, inner ear sensory epithelia formation, and HC/supporting cell differentiation. Previous work has only provided indirect evidence for the role of Sox2, using a partially uncharacterized Sox2 model with limited or no expression of Sox2 in the ear^21^,^39^ or induced delayed loss of Sox2 on HCs using inducible Sox2-creER mediated recombination^40^ or worked in chickens^13^,^15^. Our data confirm and extend previous findings, but put them for the first time on a solid experimental basis consistent with the emerging concept of Sox-mediated neurosensory precursor development regulation^2^,^41^,^42^. The inner ear changes induced by the deletion of Sox2 in our Sox2 CKO mutant are summarized in Fig. 8.

Inner ear sensory neuron formation requires a series of transcription factors starting with the expression of the bHLH genes Neurog1^7^,^24^, Neurod1^43^ and followed by several other factors needed to fully differentiate the neurons, guide their migration away from the ear, and process growth to the ear^6^,^44^. Neurons delaminate from multiple sites in the sensory epithelia or areas adjacent to the sensory epithelia such as the region between the cochlear base and the saccule^10^,^45^. Additionally, neurons delaminate from the cochlea with a large number of neurons delaminating from the apex^46^. Spiral ganglion neurons exit the cell cycle in a base to apex progression^8^. In the Sox2 CKO, all early forming vestibular neurons seem to develop normally. However, the formation of late-forming spiral ganglion neurons of the cochlear apex is absent, indicating a dependency on the continuous expression of Sox2 for their formation. Given that apical spiral ganglion neurons are among the last neurons of the ear to exit the cell cycle^47^, we suggest that neuronal precursor expansion and maintenance depends on Sox2. Since Neurog1 expression, and thus neuronal precursor specification, precedes the onset of Isl1 expression^17^,^24^ and Isl1-cre-mediated recombination by about 2 days (Supplementary Fig. 1), we presume that an earlier function of Sox2 protein is to stabilize early neuronal development by interacting with bHLH genes, as previously suggested^2^,^15^. A critical test of our hypothesis using an inducible *Sox2-creER* line was recently performed for the cochlea but the possible effects of a delayed loss of *Sox2* on ear neuronal development were not reported^40^.

The embryonic viability of sensory neurons critically depends on two neurotrophins, Bdnf and Ntf3, released from the differentiating sensory epithelia^27^,^28^. In our Sox2 CKO, the targeted innervation of vestibular neurons to the area of the cristae of the semicircular canal ampullae was initially formed even in the complete absence of target HCs (Fig. 2a,b), suggesting pathfinding along the ear^48^, possibly involving Schwann cells^29^. However, later in the development, the fibers retracted in the absence of sensory epithelia as in other mutants without cristae formation^23^. Thus, the absence of sensory epithelia results in the delayed loss of innervation. In contrast, the pattern of innervation within a sensory epithelium critically depends on the density and distribution of HCs as is very obvious in the unusual innervation pattern encountered in the base of the cochlea (Figs 2 and 3). Our data therefore suggest a biphasic loss of neurons in our Sox2 CKO: late-forming neurons never develop due to a loss of Sox2, which is required to initiate their differentiation, whereas early forming neurons die after differentiation proportionally to the reduced presence of HCs to support them. We also presume this to be true for the reported absence of neurons in *Lcc* mutants^25^. We predict that the recently reported delayed Sox2 deletion^40^ should show a comparable pattern of neuronal loss in the cochlea.

Multiple lines of research have established that Sox transcription factors are needed to maintain pluripotency, but also to initiate differentiation through the upregulation of other transcription factors, in particular bHLH factors and other Sox factors^2^,^41^, which subsequently form a negative feedback loop to suppress early Sox factors for normal differentiation^4^,^42^. Previous work has demonstrated a critical dependence of all HC development on Sox2^21^ but some sensory epithelia in addition critically depend on Sox4/11^49^. Data in the ear show a loss of Sox2 in differentiated HCs due to Atoh1 repression^39^ but also that Sox2 expression is needed for Atoh1 upregulation^40^. Our data support this, since *Atoh1* was expressed only in areas with residual Sox2 expression in the Sox2 CKO inner ear. However, for the first time, our data show that some yet to be defined level and time of Sox2 expression is needed for normal HC development. We showed that the level of *Atoh1* depends on the level of Sox2 expression. To define the spatiotemporal expression pattern of *Atoh1* and *Sox2* would require multicolor quantitative PCR to show the co-localization of *Atoh1* and *Sox2* mRNA in a given hair cell precursor over time. Our Sox2 CKO data demonstrate that different sensory epithelia vary remarkably in HC formation: some epithelia never form (cristae of the semicircular canal ampullae, apical turn of the organ of Corti), while others are variably reduced (utricle, saccule, basal turn of the organ of Corti). There are two other possibilities beyond a simple delay in *Sox2* recombination that could explain the differential loss of sensory epithelia: Isl1-cre could distinctly affect some but not other epithelia precursors, leading to their complete or partial loss. However, this possibility is effectively ruled out by our analysis of the inner ear of *Isl1-cre; Atoh1*^*f/f*^ mutants. Our data show a uniform effect of Isl1-cre in all sensory epithelia of *Isl1-cre; Atoh1*^*f/f*^ mutants with no differentiated HCs in the inner ear (Supplementary Fig. 4). Alternatively, since only epithelia known or suspected to have common neuronal/hair cell progenitors^7^,^8^,^9^ retain HCs, it might be possible that only HCs derived from such common neurosensory progenitors form, whereas epithelia without such common precursors develop no HCs at all (cristae of the semicircular canal ampullae, apical turn of the organ of Corti). The expression of neuronal markers in some of these HCs (Fig. 6) supports this notion, but lineage tracing is needed to prove this suggestion.

Previous work has demonstrated that the level and duration of Atoh1 expression driven by multiple transcription factors^42^ is crucial for HC differentiation and survival^33^,^34^,^36^,^38^. Using *in situ* hybridization, we found limited and transient expression of *Atoh1* mRNA, presumably leading to a differential loss of differentiating HCs, as previously reported^34^,^38^. We find profound variability of remaining HCs, which indicates that the compounding effect of Isl1-cre expression onset, effective recombination delay of *Sox2* and retention of Sox2 protein introduce HC specific irregularity into the differentiation process, compounded by alterations in Atoh1 expression.

Atoh1 is not only expressed in HCs but in the majority of neurons in the brain, among other cell types^35^,^50^. However, only in the ear, Atoh1 seems to control the formation of stereocilia and their organization in a dose dependent fashion^36^. Establishing the expression profile of transcription factors in the few HCs of the basal turn of our Sox2 CKO, which differentiate with some degree of normality, could provide candidate genes that can help to define the activation cascade needed to differentiate normal stereocilia needed for functional hair cells.

**Figure 7:**
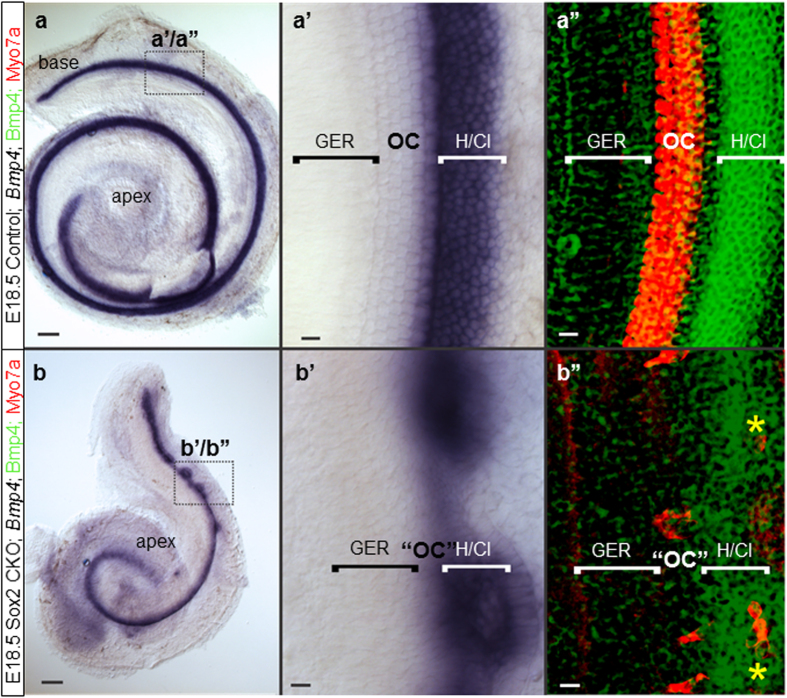
The cellular boundaries of the inner ear are changed in the Sox2 CKO. (**a,a’,b,b’**) Loss of Sox2 results in aberration of *Bmp4* expression. Instead of being separated by the organ of Corti from the GER, *Bmp4* expression is adjacent to the GER. (**b, b’,b”**) Only the base shows rings of lateral *Bmp4* expression and Myo7a positive HCs are both in the center of these rings as well as at the boundary between GER and *Bmp4* domain (b”; yellow asterisks). (**a’-a”**) In controls, the *Bmp*4 expression in Hensen/Claudius cells is always lateral to the organ of Corti. Scale bars: 10 μm except 100 μm in a and b. GER, greater epithelial ridge; H/Cl; Hensen/Claudius cells; OC, organ of Corti; “OC”, atypical organ of Corti in the mutant.

**Figure 8:**
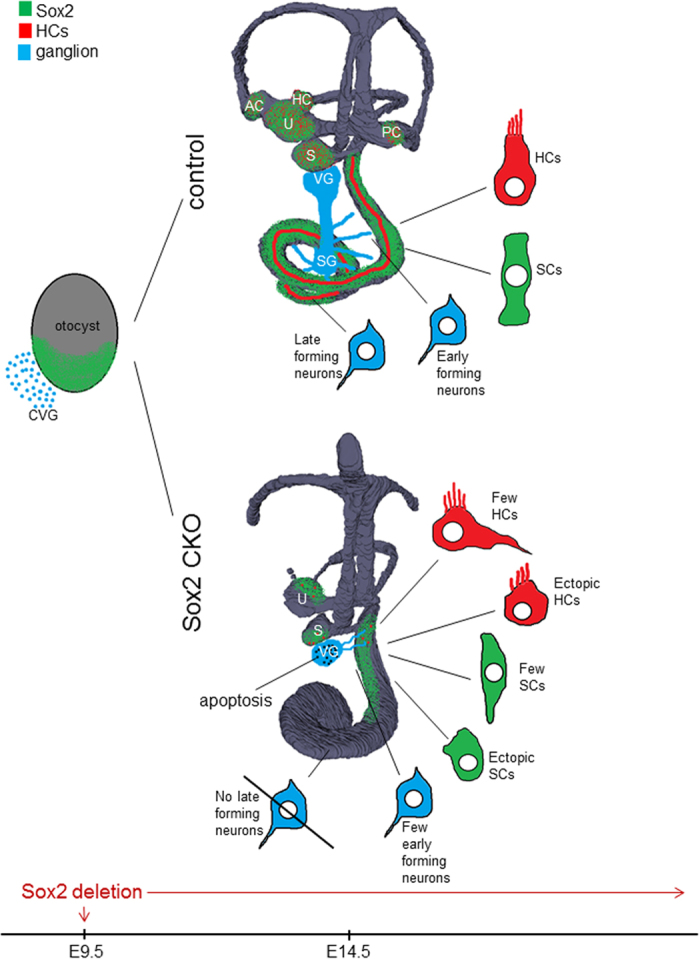
Summary of Sox2 CKO inner ear changes. *Sox2* deletion by Isl1-cre results in profound morphological changes at E14.5: all three cristae of the semicircular canal ampullae are missing, all remaining sensory organs are smaller and have a decreased size of the sensory area. Neuronal formation is eliminated in the apex of the cochlea, whereas vestibular and basal turn neurons gradually die due to limited support by the reduced sensory epithelia. Loss of innervation toward all epithelia except for the apex of the cochlea is secondary to the lack or reduced differentiation of HCs, which is either completely absent in the apex and semicircular canal cristae, or variably disabled in the base of the cochlea, utricle, and saccule. Many HCs have an unusual neurosensory phenotype. Similarly, supporting cells (SCs) have atypical features, altered expression of markers, and abnormal distribution, forming the aberrant organ of Corti. Many ectopic HCs and SCs are also found in the cochlear base. The spatial distribution of Sox2 expression is shown in green, blue color shows neurons, and red depicts HCs. AC, anterior crista; CVG, cochleovestibular ganglion; HC, horizontal crista; HCs, hair cells; PC, posterior crista; S, saccule; SCs, supporting cells; SG, spiral ganglion; U, utricle; VG, vestibular ganglion.

The organ of Corti has only two HC types but a rich variety of supporting cell types, each with unique properties and markers expressed in them^35^, forming multiple feedback loops through the expression of diffusible factors, as well as the delta-notch lateral inhibition interactions^9^ with HCs^36^. HC defects could thus result in supporting cell defects, as recently reported^36^. However, certain unusual features, such as the morphology of inner pillar cells (Fig. 4e-f”) or the unusual distribution of *Bmp4* medial to remaining HCs (Fig. 7b”), suggest that Sox2 may have a more direct role to play in cell fate execution of supporting cells that needs to be explored using supporting cell specific deletion.

In summary, our data on this novel conditional mutation of the important developmental transcription factor Sox2 show a much deeper involvement of Sox2 in neurosensory development of the ear at various levels beyond HC differentiation^21^,^40^. First, our Sox2 CKO mutant with a delayed deletion of Sox2 indicates that neuronal development depends directly on Sox2, as the latest-forming spiral ganglion neurons never form. Second, our data confirm that the differentiation of HCs depends on Sox2. Furthermore, a variability in differentiation of HCs (cell number, size, stereocilia development, incomplete segregation neuronal and HC phenotype) suggests dependency on the level and duration of Sox2 expression. Third, Sox2 deletion affects the development of supporting cells, as shown by the altered expression of Hes5 and p75, and by the variable topology and unusual shape of p75 positive inner pillar cells. Fourth, the formation of boundaries between the organ of Corti and GER in the cochlea is affected by the loss of Sox2. Selective conditional deletion of Sox2 in neurons, HCs, or supporting cells using differentially delayed cre expression is now needed to sort out the specific function of Sox2 in these cell types and to clarify questions raised by our novel mouse model.

## Methods

### Animals

This study was performed in agreement with the Guide for the Care and Use of Laboratory Animals (National Research Council. Washington, DC. The National Academies Press, 1996.). The experimental design was approved by the Animal Care and Use Committee of the Institute of Molecular Genetics, Czech Academy of Sciences. The experimental mice were housed in a controlled environment (12-h light-12-h dark cycles) with free access to food and water. All experiments were performed with the littermates cross-bred from two transgenic mouse lines: *Sox2*^*flox*^ (*Sox2*^*tm1.1Lan*^*/J*) and *Isl1-cre (Isl1*^*tm1(cre)Sev*^*/J*) from The Jackson Laboratory. Breeding pairs contain a mouse with two floxed *Sox2* alleles (*Sox2*^*f/f*^) and a mouse with one floxed *Sox2* allele together with one *Isl1-cre* allele (*Isl1*^*cre/*+^*;Sox2*^*f/*+^). Experimental mutant mice (*Isl1-cre;Sox2*^*f/*f^) survive until birth but they are not viable. The noon of the day the vaginal plug was found was designated as E0.5. Genotyping was performed by PCR on tail DNA. The annealing temperature was 55 °C for *cre* amplification and 64 °C for *Sox2* amplification. The primers and corresponding PCR products were as follows: cre forward (Fwd) (5′-GCC TGC ATT ACC GGT CGA TGC AAC GA-3′) and cre reverse (Rev) (5′-GTG GCA GAT GGC GCG GCA ACA CCA TT-3′) with 700 bp product; Sox2 Fwd (5′-TGG AAT CAG GCT GCC GAG AAT CC-3′), Sox2 Rev wild type (5′-TCG TTC TGG CAA CAA GTG CTA AAG C-3′) with 427 bp product or Sox2 Rev mutant (5′-CTG CCA TAG CCA CTC GAG AAG-3′) with 546 bp product. Additional crossing with transgenic lines the *Isl1-cre (Isl1*^*tm1(cre)Sev*^*/J*) and floxed *Atoh1*^51^ was used to generate conditional *Atoh1* knock-out mice (*Isl1-cre; Atoh1*^*f/f*^; Atoh1 CKO). Genotyping primers for *Atoh1*^*flox*^were Atoh1loxF (5′-CAG ATC CCA CAG AAG TGA CG-3′) and Atoh1loxR (5′-ACA CTG CTG GAC ACA CTT GG-3′).

### Hearing function evaluation

To assess the auditory function of the experimental animals recording of auditory brainstem responses (ABR) and distortion product otoacoustic emissions (DPOAE) was performed. All tests were carried out on anaesthetized mice as previously described^52^. Briefly, hearing thresholds were determined at 2, 4, 8, 16, 32 and 40 kHz and DPOAEs were recorded at individual frequencies over the frequency range 4–38 kHz with a resolution of four points per octave. The average values per group were calculated and the results (mean ± SD) were plotted in audiograms (ABR thresholds) and DP-grams (DPOAEs).

### Histology of the inner ear

Embryos were dissected in cold PBS and fixed in 4% paraformaldehyde.

#### 3D reconstruction

Inner ears were dehydrated in ethanol series and stained with 0.5 μg/ml Rhodamine B isothiocyanate in 100% ethanol^53^. Samples were then cleared by MSBB solution and mounted onto a glass slide prior to imaging. Images were taken by Zeiss LSM 5 DUO or Leica SPE confocal microscopes and processed in ImageJ^54^. 3D structures of inner ears were reconstructed from confocal stacks in 3D Slicer by manually segmenting areas of interest.

#### Scanning electron microscopy

All of the excessing structures and membranes were removed from the sensory organs. Samples in porous specimen pots were extensively washed and dehydrated through an alcohol series followed by absolute acetone. Tissues were critical point dried in liquid CO_2_ in a K 850 unit (Quorum Technologies Ltd, Ringmer, UK). The dried samples were mounted onto carbon conductive double sided adhesive discs and sputter-coated with 20 nm of gold in a Polaron Sputter-Coater (E5100) (Quorum Technologies Ltd, Ringmer, UK). The final samples were examined in a FEI Nova NanoSem 450 scanning electron microscope (FEI Czech Republic s.r.o.) at 5 kV using a secondary electron detector.

#### X-gal staining

The mouse line *Isl1-cre* was bred with R26R-*lacZ (Gt(ROSA)26Sor*^*tm1Sor*^, Jackson Laboratory) and animals carrying both loci were subjected to X-gal staining. Fixed tissues were washed in detergent solution (0.1 M phosphate buffer (pH 7.3), 2 mM MgCl2, 0.01% sodium deoxycholate, 0.02% IGEPAL CA-630) and then incubated at 37 °C in X-gal staining solution (1 mg/ml X-gal, 5 mM potassium ferrocyanide, 5 mM potassium ferricyanide, 20 mM TrisCl (pH 7.3). Samples were washed in PBS with 25 mM EDTA and imaged by a Nikon SMZ dissection microscope.

#### Immunohistochemistry

For vibratome sections, samples were embedded in 4% agarose gel and sectioned at 80 μm on a Leica VT1000S vibratome. Vibratome sections, whole inner ears or whole embryos were defatted in 70% ethanol and then rehydrated and blocked with serum. Samples were then incubated with primary antibodies. The primary antibodies used were anti-Sox2 (1:500, #AB5603, Millipore or 1:250, #sc-17320, Santa Cruz Biotechnology), anti-Islet1/2 (1:200, #39.4D5, DSHB), anti-Islet1 (1:130, #39.3F7, DSHB) anti-Myo7a (1:500, #25-6790, Proteus Biosciences Inc), anti-acetylated Tubulin (1:400, #T6793, Sigma), anti-cre (1:500, #908001, BioLegend), anti-Cleaved Caspase 3 (1:100, #9661, Cell Signaling), anti-Neurofilament 200 (1:200, #N4142, Sigma-Aldrich) and anti-p75 (1:1000, #N3908, Sigma-Aldrich). Anti-Isl1/2 (#39.4D5) and anti-Isl1 (#39.3F7) antibodies developed by Jessell, T.M./Brenner-Morton, S. were obtained from the Developmental Studies Hybridoma Bank, created by the NICHD of the NIH and maintained at The University of Iowa, USA. Secondary antibodies used were Alexa Fluor 488 (#115-545-146, Jackson Immuno Research), Alexa Fluor 594 (#111-585-144, Jackson Immuno Research), DyLight 488 (#205-485-108, Jackson Immuno Research) and DyLight 594 (#205-515-108, Jackson Immuno Research). Samples were counterstained with Hoechst nuclear stain. Samples were mounted on slides in Polymount or Antifade medium and analyzed by Zeiss LSM 5 DUO, Zeiss LSM 880 or Leica SPE confocal microscopes. Images were processed in ImageJ. Individual cochleae were flat-mounted with the sensory epithelium facing up. The entire length of the cochlear duct from the hook region along the basilar membrane was measured by ImageJ. Myo7a positive HCs were quantified after whole mount immunostaining using LAS AF Lite draw counter. The total number of HCs was determined in the entire utricle and saccule, and in the entire Sox2 CKO cochlea. The number of HCs in the control cochlea represents the total number of HCs in 1.5 mm of the base of the cochlea. Caspase3 positive cells were quantified in the vestibular ganglion at E11.5-E13.5 and in the superior vestibular ganglion at E14.5-E15.5 in both control and Sox2 CKO mice after the whole mount immunostaining with the anti-Caspase3 antibody. The statistical significant differences between control and Sox2 CKO mice were analyzed by Student’s *t* test (significance assigned at the P < 0.05 level; GraphPad, 2005, USA).

#### *In situ* hybridization

*In situ* hybridization was performed using a RNA probe labeled with digoxigenin as previously described^14^. Plasmids containing cDNAs [gifts from H. Zoghbi (Atoh1), D. Wu (BMP4), A. Groves (Hes5), K Cheah (Sox2), D. Ornitz Fgf10)] were used to generate the RNA probe by *in vitro* transcription. After being anesthetized with 2,2,2 tribromoethanol (Avertin), mice were perfused in 4% paraformaldehyde (PFA) and fixed overnight in 4% PFA. The ears were dissected in 0.4% PFA and dehydrated and rehydrated in graded methanol series and then digested briefly with 20 μg/ml of Proteinase K (Ambion, Austin, TX, USA) for 15–20 minutes. The samples were then hybridized overnight at 60 °C to the riboprobe in hybridization solution. The samples were incubated overnight with an anti-digoxigenin antibody after washing off the unbound probe (Roche Diagnostics GmbH, Mannheim, Germany). After a series of washes, the samples were reacted with nitroblue phosphate/5-bromo, 4-chloro, 3-indolil phosphate (BM purple substrate, Roche Diagnostics, Germany) which is enzymatically converted to a purple colored product. The ears were mounted flat in glycerol and viewed in a Nikon Eclipse 800 microscope using differential interference contrast microscopy and images were captured with Metamorph software. The ears of the littermate of different genotype for the same gene expression were performed in the same reaction tubes to maintain the reaction accuracy.

### Lipophilic Dye Tracing

We studied the pattern of innervation in whole or dissected ears using lipophilic dye tracing in aldehyde fixed tissue as previously described^55^. Briefly, we inserted filter strips loaded with differently colored lipophilic dyes into the cochlear/vestibular nuclei of the brainstem around rhombomere 5 to label afferents and into rhombomere 4 near the midline to label facial motoneurons/efferents to the ear^56^. After appropriate diffusion time of the lipophilic tracer, we prepared the ears as whole mounts removing the lateral wall of the otic capsule, mounted with glycerol on a glass slide using appropriate spacers to avoid distortion and imaged using a Leica SP5 confocal microscope. Image stacks were collected and single images or sets of the stacks were obtained to provide detailed information about the progressive development of the ear innervation and loss over time. Selected ears were further dissected to reveal the detailed innervation of flat mounted sensory epithelia. Images were compiled into plates to show the most pertinent details using Corel Draw. Only general image modifications such as contrast or brightness adjustments were used to enhance the visual appeal without affecting the scientific content. All material was after imaging used for either *in situ* hybridization or immunological studies as previously described^57^.

## Additional Information

**How to cite this article**: Dvorakova, M. *et al*. Incomplete and delayed Sox2 deletion defines residual ear neurosensory development and maintenance. *Sci. Rep.* **6**, 38253; doi: 10.1038/srep38253 (2016).

**Publisher's note:** Springer Nature remains neutral with regard to jurisdictional claims in published maps and institutional affiliations.

## Supplementary Material

Supplementary Information
