# Supplementary material for: Incomplete and delayed Sox2 deletion defines residual ear neurosensory development and maintenance
Source: Sci Rep. 2016 Dec 5;6:38253. doi: 10.1038/srep38253 (PMC5137136; doi:10.1038/srep38253)
Supplement: Supplementary Information [file srep38253-s1.pdf]

Supporting Information

Supplementary Figures

Incomplete and delayed Sox2 deletion defines residual ear neurosensory development and maintenance

Martina Dvorakova[1,4†], Israt Jahan[3†,] Iva Macova[1,4], Tetyana Chumak[2], Romana Bohuslavova[1], Josef Syka[2], Bernd Fritzsch[3#], Gabriela Pavlinkova[1#*]

[1]Institute of Biotechnology CAS, Prague, Czech Republic

[2] Institute of Experimental Medicine CAS, Prague, Czech Republic

[3] Department of Biology, University of Iowa, Iowa City, IA, USA

[4]Faculty of Science, Charles University, Prague, Czech Republic

[†]Indicates equal contribution.

[#]indicates co-senior author

*Correspondence to: Dr. Gabriela Pavlinkova

Laboratory of Molecular Pathogenetics
Institute of Biotechnology CAS, Prague, Czech Republic
Email: gpavlinkova@ibt.cas.cz

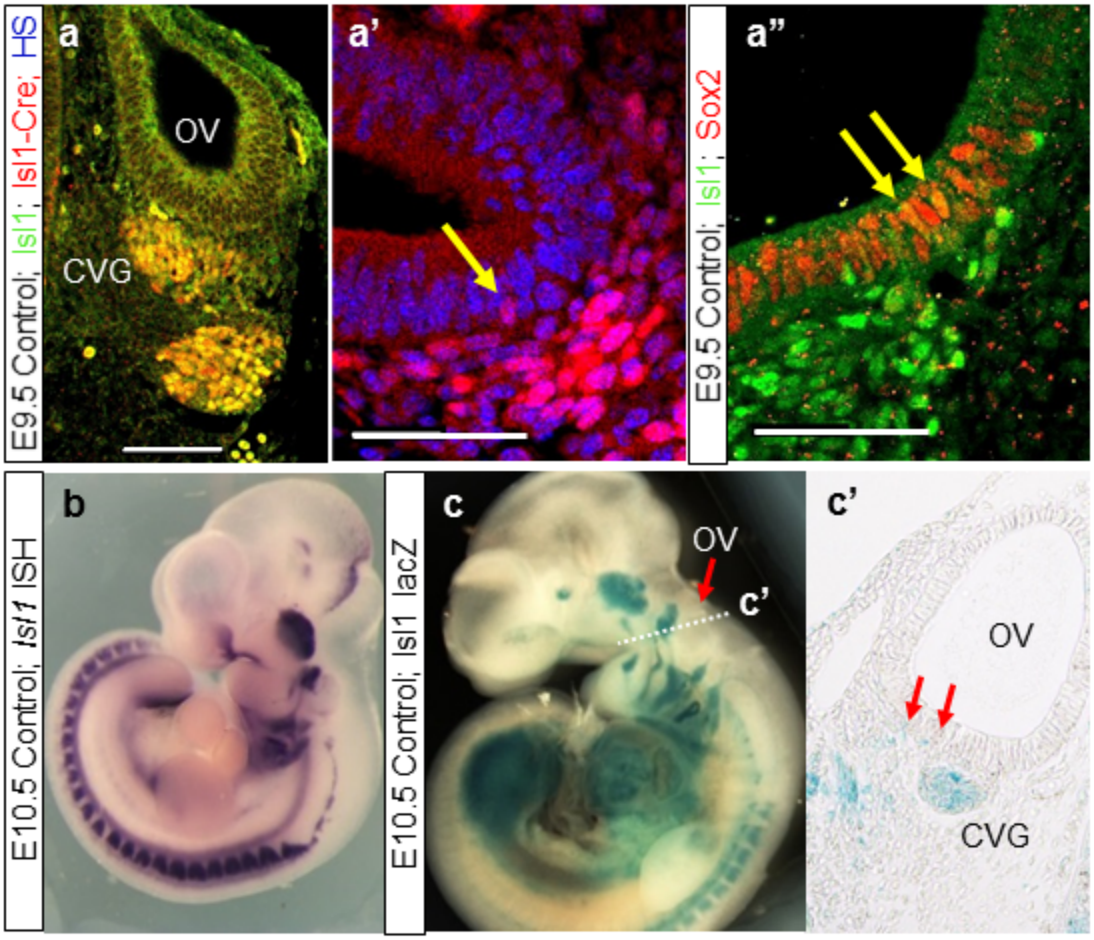

Supplementary Figure 1. Isl1-cre recombination corresponds to the expression of *Isl1* in the otocyst. (**a**) At E9.5, the expression of Isl1-cre correlates with the expression of Isl1 protein (green). (**a'**) Isl1-cre (red) is detected in the delaminated cochleovestibular (CVG) neurons and also within the otic epithelium (arrow). (**a''**) Isl1 (green) is co-expressed with Sox2 (red) in the otic neuroepithelium at E9.5 (arrow). (**b**) *Isl1* mRNA is expressed in the progenitor cells of sensory epithelium and in otic ganglion at E10.5. Cre-mediated β-gal reporter expression shows Cre recombination in the tissues corresponding to the $Isl1^{+}$ domain in E10.5 embryos (**c, c'**). OV, otic vesicle; CVG, cochleovestibular ganglion. Scale bar: 100 μm.

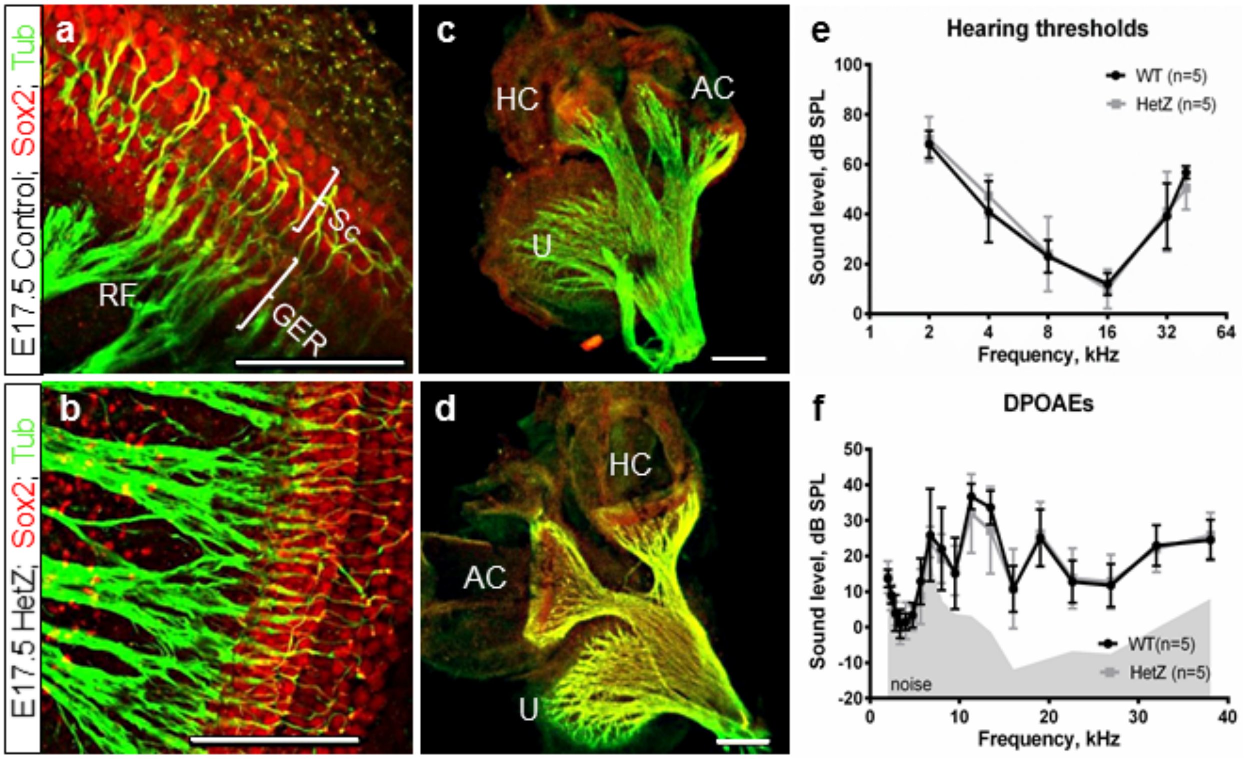

Supplementary Figure 2. The development of the inner ear sensory organs of heterozygous (Hetz) *Isl1-cre/+;Sox2$^{f/+}$* mutant is not altered. (**a-d**) Sox2 expression (red) and the pattern of innervation (tubulin, green) in the sensory organs of the inner ear is comparable between heterozygous *Isl1-cre/+;Sox2$^{f/+}$* (HetZ) and wild type littermate controls. (**e, f**) Hearing functions of HetZ are similar compared to wild type littermates as shown by hearing thresholds (ABRs) and deteriorated distortion product otoacoustic emissions (DPOAEs). Results of hearing tests of 1 month old mice are presented as means ± SD. Scale bars: 50 µm (a, b), 100 µm (c, d). AC, anterior crista; HC, horizontal crista; GER, greater epithelial ridge; RF, radial fibers; Sc, supporting cells; U, utricle.

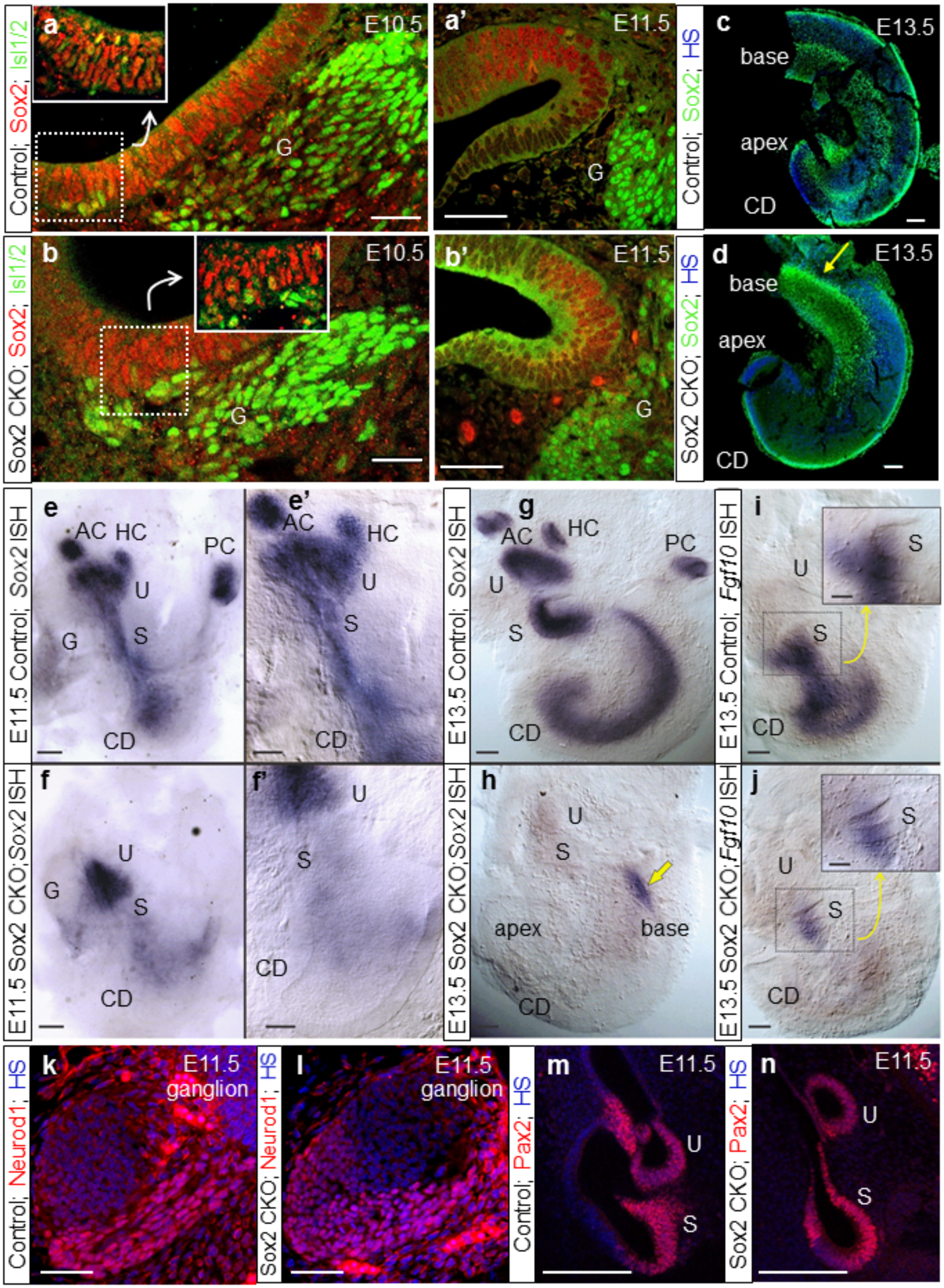

Control; Sox2; Isl1/2
a
E10.5
G
a'
E11.5
G
Control; Sox2; HS
c
E13.5
base
apex
CD
Sox2 CKO; Sox2; Isl1/2
b
E10.5
G
b'
E11.5
G
Sox2 CKO; Sox2; HS
d
E13.5
base
apex
CD
E11.5 Control; Sox2 ISH
e
AC
HC
PC
U
S
G
CD
e'
AC
HC
U
S
CD
E13.5 Control; Sox2 ISH
g
AC
HC
PC
U
S
CD
E13.5 Control; Fgf10 ISH
i
U
S
S
CD
E11.5 Sox2 CKO;Sox2 ISH
f
U
G
S
CD
f'
U
S
CD
E13.5 Sox2 CKO;Sox2 ISH
h
U
S
apex
base
CD
E13.5 Sox2 CKO;Fgf10 ISH
j
U
S
S
CD
Control; Neurod1; HS
k
E11.5
ganglion
Sox2 CKO; Neurod1; HS
l
E11.5
ganglion
Control; Pax2; HS
m
E11.5
U
S
Sox2 CKO; Pax2; HS
n
E11.5
U
S

Supplementary Figure 3. Gene expression pattern is effectively altered in Sox2 CKO mutants. Reduced overlap of Isl1 and Sox2 immunostaining is apparent in Sox2 CKO ears at E10.5 (**a**, **b**). Some Sox2 protein can still be detected in the inner ear up to E11.5 (**a'**, **b'**). At E13.5, Sox2 is expressed only in the base of the Sox2 CKO cochlea, whereas in the controls, the expression of Sox2 (green) occurs throughout the entire length of the cochlea (**c**, **d**). The *Sox2* mRNA reduction is apparent in the utricle, saccule and cochlear base at E11.5 with no expression in the semicircular canal cristae and in the apical half of the growing cochlear duct (**e**-**f'**). At E13.5, there is only a weak expression of *Sox2* mRNA in the base of the cochlea compared to controls (**g**, **h**). No *Fgf10* mRNA could be detected in canal cristae and only limited *Fgf10* labeling is in saccular neurons of the Sox2 CKO (**i**, **j**). The expression of Neurod1 in the otocyst-derived ganglion neurons is comparable between Sox2 CKO and controls at E11.5 (**k**, **l**). The expression of Pax2 appears unaffected in the Sox2 CKO inner ear compared to controls at E11.5 (**m**, **n**). Scale bars: 100 µm, except a and b that indicates 50 µm. AC, anterior crista; CVG, cochleovestibular ganglion; CD, cochlear duct; G, ganglion; HC, horizontal crista; PC, posterior crista; U, utricle; S, saccule.

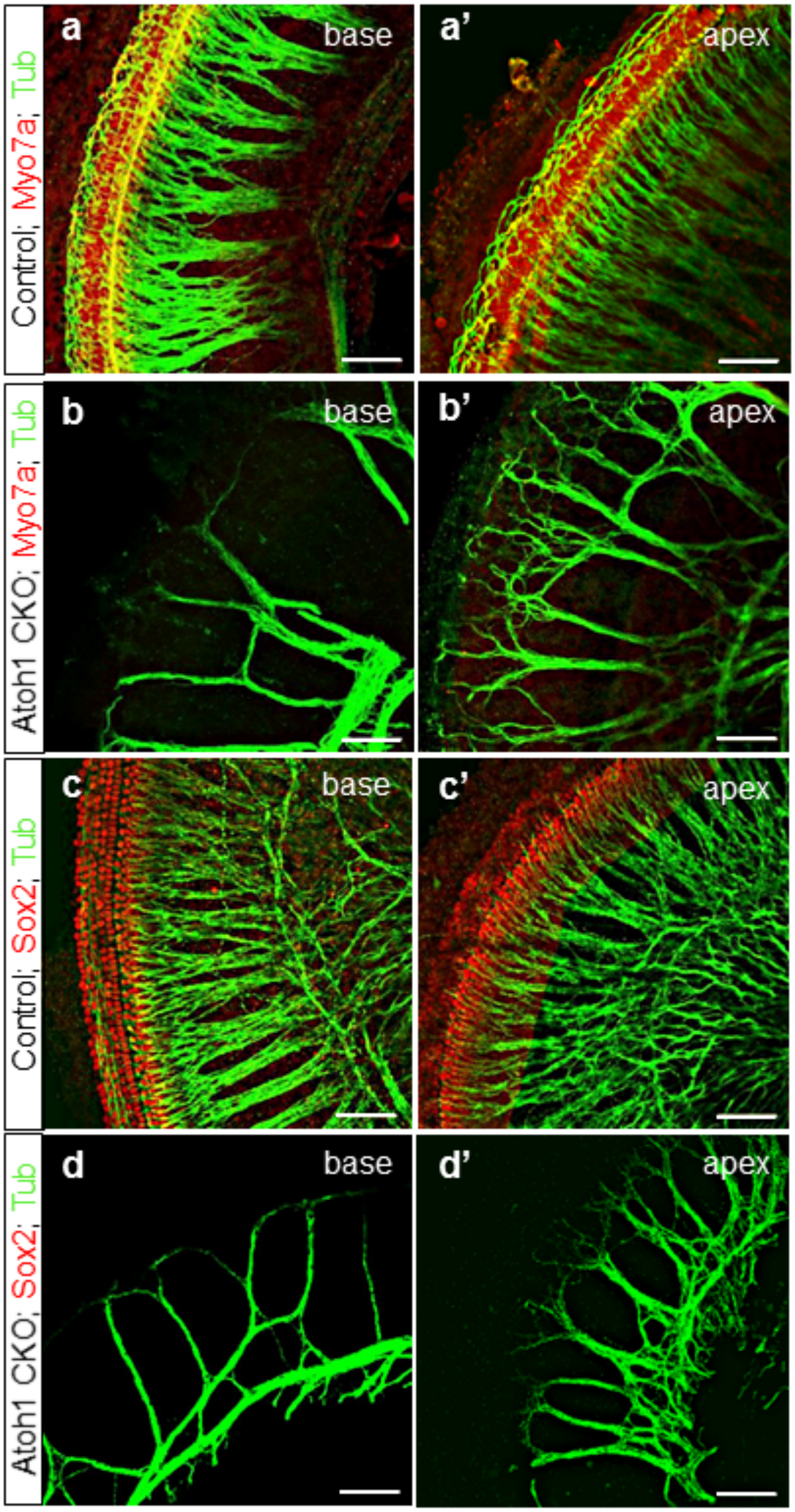

Supplementary Figure 4. *Isl1-cre;Atoh1$^{f/f}$* (Atoh1 CKO) mutants show a complete loss of all HCs and Sox2 expression. Isl1-cre mediated recombination of floxed *Atoh1*

results in a complete loss of HCs in the cochlear base (**a, b**) and apex (**a', b'**), as indicated by the expression of HC marker, Myo7a. These data show a uniform action of Isl1-cre along the entire cochlea. Our analysis of Isl1-cre-mediated β-gal reporter expression showed cre recombination in the organ of Corti (OC) as well as in the greater epithelial ridge (GER) in the cochlea at P0. Accordingly, there is a complete loss of Sox2 expression in OC and GER of Atoh1 CKO mice (**c, c', d, d'**), suggesting that the absence of Atoh1 or/and the organ of Corti causes the loss of Sox2 expression in GER and supporting cells in the cochlea. Thus, neither the base nor the apex of these mutants show any Myo7a positive HCs (**b, b'**), suggesting that incomplete recombination of *Sox2* by Isl1-cre is not the explanation of the patchy retention of some Myo7a positive cells in *Isl1-cre;Sox2*$^{f/f}$ mice. Scale bar, 50 μm.

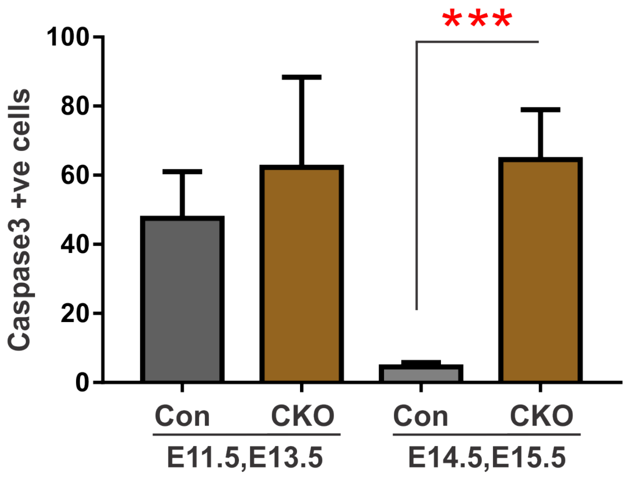

Supplementary Figure 5. Caspase-3 mediated neuronal death massively progresses in the Sox2 CKO inner ear after E13.5 compared to controls. The total number of Caspase3^{+} cells was determined in the vestibular ganglia (E11.5-E13.5) and superior vestibular ganglia (E14.5 and E15.5) of the control (Con) and Sox2 CKO ear at indicated embryonic days. Caspase3 positive cells were quantified after whole mount immunostaining with anti-Caspase3 antibody using LAS AF Lite draw counter. The values represent means ± SD (N = 4 individuals/group). ***P<0.0001, *t*-test.

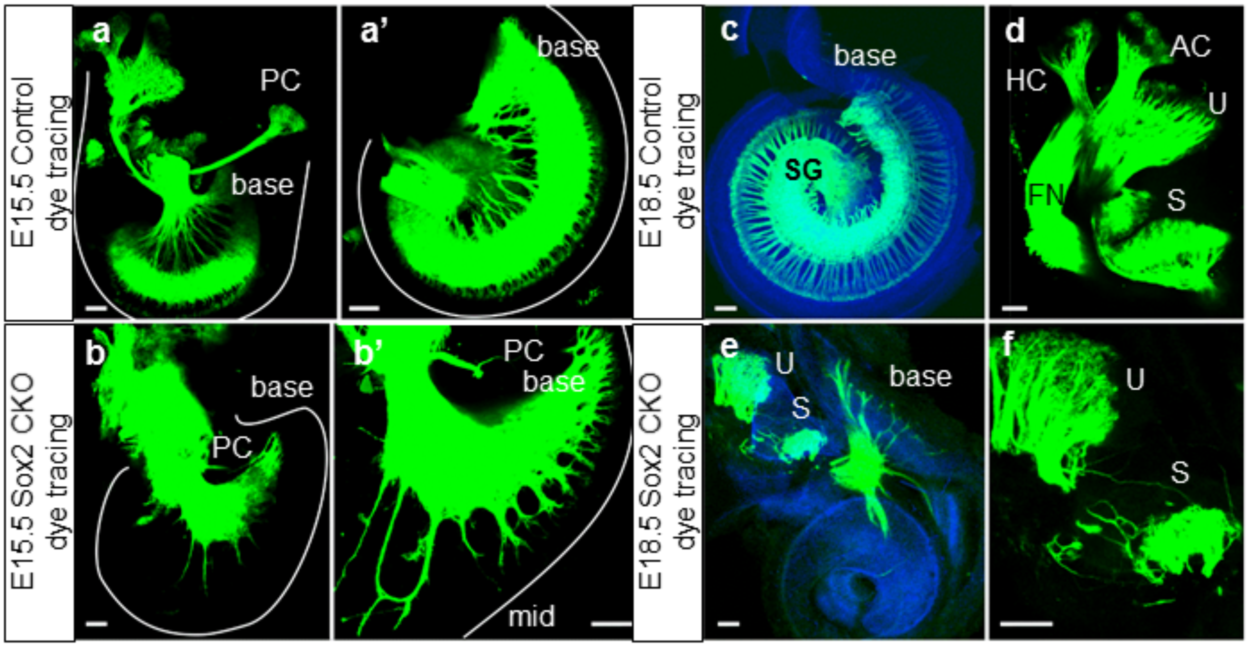

Supplementary Figure 6. The pattern of innervation in the inner ear is profoundly changed in Sox2 CKO mutants. The pattern of innervation differs dramatically between control and mutant littermates at E15.5 (**a, b**) and E18.5 (**c-f**). Spiral ganglion neurons (SG) are present only in the basal turn in mutants (**a, a', b, b'**) with only few fibers extending toward the middle turn (**a', b'**). The posterior vertical canal crista (PC) receives a prominent fiber track in control mice (**a**) but only few fibers extend some distance toward this epithelium in mutants (**b, b'**). Due to further reduction of SG by apoptosis only a small population remains near the basal tip of the cochlea in the mutant (**c, e**) and only limited innervation remains to the saccule (**d-f**). AC, anterior canal crista; HC, horizontal canal crista; PC, posterior canal crista; S, saccule; SG spiral ganglion neurons; U, utricle. Scale bars, 100 µm.

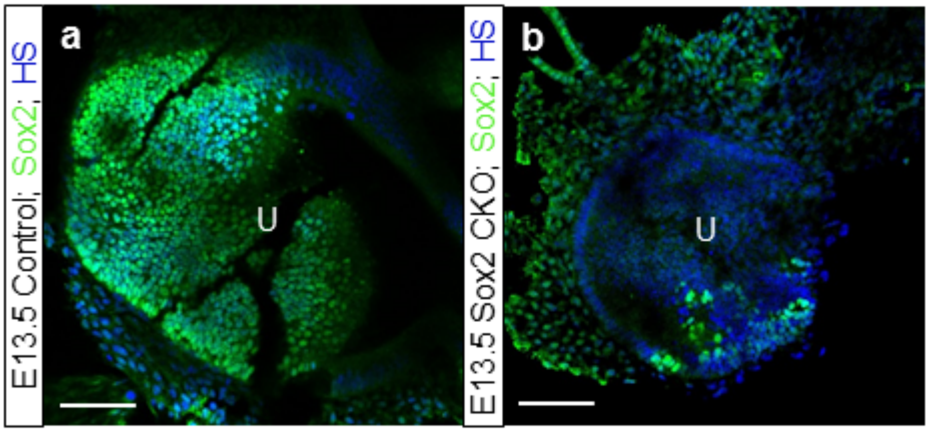

Supplementary Figure 7. Delayed deletion of Sox2 by Isl1-Cre leaves some Sox2⁺cells in the utricle. Compared to controls (**a**), the size of Sox2⁺ domain is substantially decreased in the Sox2 CKO utricle (**b**). U, utricle. Scale bars, 100 μm.

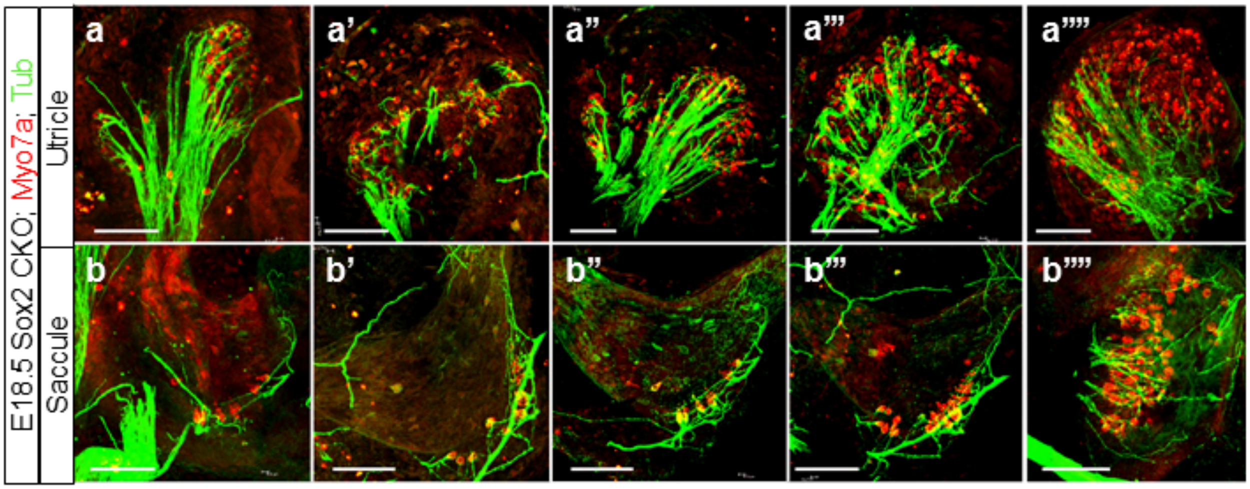

Supplementary Figure 8. Variation of HC numbers is detected in the Sox2 CKO. Numbers of HCs and their distribution in the utricle (**a-a''''**) and saccule (**b-b''''**) vary between individual animals but are overall dramatically reduced compared to littermate controls. Scale bars, 100 μm.

Table S1. Quantification of HCs in the utricle, saccule and cochlea of E18.5 control and Sox2 CKO mice.

	Control Utricle	Sox2 CKO Utricle	Control Saccule	Sox2 CKO Saccule	Control Cochlea*	Sox2 CKO Cochlea
	1,031	132	880	15	908	21
	1,068	247	910	59	774	42
	978	112	1,190	28	719	31
	1,005	197	817	12	871	22
		88		94	774	36
		334		95	717	28
					809	
Average	*1,021*	*185*	*949*	*51*	*796*	*30*
SD	*38*	*94*	*165*	*38*	*72*	*8*

Myo7a positive HCs are quantified after whole mount immunostaining using LAS AF Lite draw counter to avoid counting error. The total number of HCs was determined in the entire utricle and saccule, and in the entire Sox2 CKO cochlea. *The number of HCs in the control cochlea represents the total number of HCs in 1.5 mm of the base.
